# Frailty in older people living with HIV: current status and clinical management

**DOI:** 10.1186/s12877-022-03477-7

**Published:** 2022-11-30

**Authors:** D. Scott Kehler, Jovana Milic, Giovanni Guaraldi, Tamas Fulop, Julian Falutz

**Affiliations:** 1 Division of Geriatric Medicine, Department of Medicine, Dalhousie University, Halifax, NS Canada; 2 School of Physiotherapy, Faculty of Health, Dalhousie University, Room 402 Forrest Building 5869 University Ave, B3H 4R2, PO Box 15000 Halifax, NS Canada; 3 Department of Surgical, Medical, Dental and Morphological Sciences, University of Modena and Reggio Emilia, Modena, Italy; 4 Department of Medicine, Geriatric Division, Research Center On Aging, Université de Sherbrooke, Sherbrooke, QC Canada; 5 Division of Geriatric Medicine, Division of Infectious Diseases, Comprehensive HIV Aging Initiative, McGill University Health Center, Montreal, QC Canada

**Keywords:** HIV, Frailty, Sarcopenia, Pathophysiology, Transitions, Resilience

## Abstract

This paper will update care providers on the clinical and scientific aspects of frailty which affects an increasing proportion of older people living with HIV (PLWH). The successful use of combination antiretroviral therapy has improved long-term survival in PLWH. This has increased the proportion of PLWH older than 50 to more than 50% of the HIV population. Concurrently, there has been an increase in the premature development of age-related comorbidities as well as geriatric syndromes, especially frailty, which affects an important minority of older PLWH. As the number of frail older PLWH increases, this will have an important impact on their health care delivery. Frailty negatively affects a PLWH’s clinical status, and increases their risk of adverse outcomes, impacting quality of life and health-span. The biologic constructs underlying the development of frailty integrate interrelated pathways which are affected by the process of aging and those factors which accelerate aging. The negative impact of sarcopenia in maintaining musculoskeletal integrity and thereby functional status may represent a bidirectional interaction with frailty in PLWH. Furthermore, there is a growing body of literature that frailty states may be transitional. The recognition and management of related risk factors will help to mitigate the development of frailty. The application of interdisciplinary geriatric management principles to the care of older PLWH allows reliable screening and care practices for frailty. Insight into frailty, increasingly recognized as an important marker of biologic age, will help to understand the diversity of clinical status occurring in PLWH, which therefore represents a fundamentally new and important aspect to be evaluated in their health care.

## Introduction

The availability of effective, better tolerated, and more convenient combination antiretroviral therapy (cART) has resulted in the increased survival of people living with HIV (PLWH). Their longevity is currently almost the same as that of the general population [1, 2]. However, although many PLWH are clinically well, the health state of PLWH has changed and typical age-related co-morbidities occur more frequently and possibly sooner, often from 5–10 years earlier [3]. Furthermore, conditions referred to as geriatric syndromes are also increasingly diagnosed in some PLWH [4, 5]. Among these, the condition of frailty is an important additional outcome among PLWH, along with recommendations to screen, assess and ideally reverse this condition [6]. This review will focus on the role of frailty in the overall management of older PLWH (OPLWH).

Frailty was initially described in the insurance industry over 40 years ago as a measure of the heterogeneity of mortality risk of people over 65 [7]. The term was then used to describe either dependent, institutionalized persons over 65 [8] or hospitalized older patients with multimorbidity and an increased mortality risk [9]. This concept has evolved and frailty is now described in physical, cognitive, social, emotional, and economic domains [10]. Overall, frailty represents a state of vulnerability to adverse health outcomes occurring in response to a wide variety of stressors. Although frailty was originally described among community-living populations over 65 years old, frailty has been studied over the past several decades in other populations and settings, including, but not limited to, surgical patients, end-stage liver pre-transplant, hemodialysis, diabetes, chronic inflammatory conditions, long-term care, and otherwise healthy community living adult populations. Large surveys of frailty in the general UK population have been performed, and showed that the prevalence from age 30–60 was 3–5%. This increased after the age of 60, reaching 25–30% after age 80 [11]. Frailty is shown to be more important than either chronologic age or comorbidities in predicting survival in patients admitted with Covid-19 complications [12]. Mouse models of frailty have proven useful to the investigation of contributing biologic conditions [13].

As the overall survival of PLWH has increased, the median age in high-income countries is now in the mid-50’s with projections for the proportion of those over 65 to increase significantly in the next 10 years [14]. Geriatric syndromes including frailty, cognitive impairment, sarcopenia, falls and impaired mobility, sensory impairment, and polypharmacy occur frequently and earlier in OPLWH [4, 5]. Diagnosing frailty is important as it is an independent risk factor for developing new chronic conditions, falls, cognitive decline, polypharmacy, hospitalization, loss of independence and increased mortality [15]. The earlier onset of age-related co-morbidities and geriatric syndromes lead to aging trajectories referred to as either accelerated, wherein HIV accelerates processes of aging, or accentuated, where HIV is an additional factor increasing the risk of developing comorbidities [16]. Evidence to support both processes in PLWH have been demonstrated [17, 18].

Driven largely by the changing clinical profile, the routine care of OPLWH has evolved from the focus on immuno-virologic control to an interdisciplinary approach which draws on the models of care successfully used for older patients [19–21]. Programs describing the introduction of specialized services for OPLWH have recently been published [22]. As many OPLWH are robust, it is not necessary to assess all OPLWH for frailty and screening for frailty is an important early step in the management of OPLWH. It is an accepted dictum in geriatrics that people age at different rates and age alone is a poor indicator of risk of aging-related complications. This heterogeneity in outcomes and functional status is represented by the concept of biologic age in contrast to chronologic age [23]. Several surrogate markers of biologic age are being intensely investigated, and evidence supports frailty as a possible candidate [24].

### Diagnosing frailty in PLWH

Recently, the European AIDS Clinical Society (EACS) Guidelines recommend that all PLWH over 50 be screened annually for frailty [25]. However, its implementation has been hampered for several reasons, including lack of consensus among geriatricians regarding which frailty measure, among many available, is reliable and simple enough to use in routine clinical settings [26]. Among the general older population, most studies of frailty use one of two well-known measures, namely the Frailty Phenotype (FP) [27] or the Frailty Index (FI) [28]. Each has been validated in diverse settings and populations, although the level of agreement between them is low [29]. The two metrics represent fundamentally different concepts of frailty [30–32]. The FP assumes that frailty is a distinct underlying biological syndrome, while the FI describes frailty as a state related to the accumulation of diverse age-associated health deficits [33].This has led to the recommendation that different frailty measures may be appropriate in specific settings [34]. See Table 1 for a list of popular frailty measures that have been either adopted or supported for frailty screening or assessment in PLWH.

**Table 1:** Frailty measures assessed in people living with HIV

I**ndex**	**Components**	**Diagnosis of frailty**	**Required equipment**
Frailty Phenotype (FP)	Physical frailty-based metric: weight loss, low physical activity, exhaustion, slowness, weakness (diagnostic adaptations possible)	Non-frail = 0 parameters Pre-frail = 1–2 parameters Frail = ≥ 3 parameters	Dynamometer; Stopwatch
Frailty Index (FI)	Percent diagnosed from minimum of 30 health deficits of broad origin, scored as absent (= 0) or present (= 1)	A continuous score. Frailty cut-off suggested > 0.23 (validated in community living > 65 years old)	None
Edmonton Frailty Scale (EFS)	Multidimension: cognition, mobility, comorbidities, medications, hospitalisation, social support, nutrition, mood, function, continence	Frailty = scores ≥ 7 (maximum of 17)	Stopwatch
FRAIL Scale (FRAIL)	Fatigue, resistance, ambulation, illness, loss of weight	Non-frail = 0 parametersPre-frail = 1–2 parametersFrail ≥ 3 parameters	None
Clinical Frailty Scale (CFS)	Visual and written analogue chart: frailty status related to disabilities, 9 grades: 1 = very fit; 9 = terminally ill	Non-frail = 1–4 Pre-frail = 5–6 Frailty ≥ 6	None

Time to complete most measures is about 5–20 min. FP takes the longest

#### Frailty phenotype

The FP is the most common measure cited in the literature, due to its apparent straight-forward and consistent use of five specific parameters. These include self-reported weight loss, self-reported exhaustion, low levels of physical activity as measured by a standardized questionnaire, measured 15 feet walk time, and measured grip strength [27]. The presence of 3 or more factors defines frailty, with 1–2 denoting a pre-frail state and the absence of any being considered as non-frail or robust. The frailty phenotype is considered as a pre-disability state, possibly representing a state of primary frailty, although comorbidities and disability often occur concurrently. Using the FP model, about 5–8% of community dwelling persons over 65 years old are frail, increasing to 20–25% for those who are 80 years or older [27].

In a cross-sectional analysis of the Dutch AGEhIV cohort, PLWH with a mean age of 52, had a higher prevalence of the frailty phenotype (10% vs 3% in controls) compared to HIV-negative persons with similar risk characteristics [35]. In a further study of this cohort, frailty was predictive of both incident comorbidity and mortality, independent of traditional risk factors such as age, comorbidity burden, and tobacco or alcohol use [36].

#### Frailty index

Frailty has also been described as a state resulting from the accumulation of various health deficits, which may include co-morbidities and disability. The commonly used measure applying this cumulative deficit approach is the FI, which is calculated as the proportion of health deficits that an individual has out of at least 30 assessed health-related variables. The information required to determine the FI may be generated from a variety of sources, including routine clinical signs or symptoms, confirmed co-morbidities, laboratory and imaging data, functional impairments, and psychosocial conditions [37]. Importantly, the specific deficits included in the FI can vary according to the data available. These need not be the same in different sites but must be consistently applied in an individual clinic especially with routine follow-up [38]. Although the FI was developed as a continuous measure, in the geriatric population an FI above 0.25 generally defines frailty, with different cut-points denoting non-frail, pre-frail and severely frail states. An FI greater than 0.7 is generally associated with an imminent and fatal prognosis [39]. The prevalence of frailty diagnosed using the FI metric is greater than that obtained with the FP measure, increasing from > 20% above age 65 to more than 40% in those over 80 years old [40].

In treated, clinically stable PLWH, the mean FI is 0.3. Interestingly, the FI was similar in 2 separate studies of asymptomatic, immunovirologically controlled Italian and Canadian PLWH with different mean ages of 46 and 59 respectively [41, 42].

In a comparative study conducted at the Modena HIV Metabolic clinic (MHMC), persons diagnosed as frail by both the FP or FI models displayed similar clinical characteristics, but the FI had a stronger association with age, nadir CD4, comorbidities, falls, and disability in comparison to the FP [6].

#### Other frailty metrics

As noted, other measures are available to diagnose frailty, including several that have also been studied in PLWH (Table 1). The Clinical Frailty Scale (CFS) is a validated, easy to perform, 9-point visually accessible metric which reflects a person’s degree of independence or the progressive need for assistance with activities of daily living (ADLs) or instrumental activities of daily living (IADLs). The stages range from fully independent to terminally ill [43]. It is reliable, user-friendly, requires no tools, predicts adverse outcomes, and can be determined retrospectively either by evaluating the individual or interviewing their regular caregivers. It compares favourably to the FP and FI in a study of OPLWH [42].

The Edmonton Frail Scale (EFS) is an index containing 10 domains including various health attributes, need for social support, cognition, level of dependence and functional status [44]. It has also been compared to the FP and FI in OPLWH [45]. Both the CFS and EFS describe persons as non-frail, pre-frail and frail.

Although not yet evaluated in PLWH, the simple, 5 component FRAIL Scale (Fatigue, Resistance [ability to climb 1 flight], Ambulation [ability to walk 1 block], Illnesses [> 5], and Loss of Weight [> 5%]) [46] has been recommended to be used as a screening tool for frailty in PLWH [25]. The Veterans Aging Cohort Study Index (VACS-I) was initially developed as a predictor of all-cause mortality in PLWH using both HIV-related and key, non-HIV related common laboratory parameters. It has also been studied as a frailty screening tool showing construct and predictive validity [47].

### Pathophysiology of frailty in PLWH

Frailty is fundamentally characterized biologically by age-related dysregulation of multiple physiological systems resulting in the progressive inability to maintain homeostasis in response to stressors. The key dysregulated physiological systems include the stress response (neuro-immuno-endocrine system), metabolic (insulin and mitochondrial metabolism) and musculoskeletal functioning [48–51]. These are normally highly interconnected networks. The biological drivers underlying this dysregulation include the well-known cellular and molecular hallmarks of aging and have been highlighted in a review: genomic instability, telomere attrition, epigenetic alterations, proteostasis loss, dysregulated nutrient sensing, mitochondrial dysfunction, cellular senescence, stem cell exhaustion and altered intercellular communication [52].

Whereas healthy aging relies on the integrated functioning of the normally regulated, multicomponent and interrelated parts of the system, frailty can be considered clinically as a syndrome [53], occurring when the extent of dysregulation crosses a certain threshold of mal-functioning [54]. Clinically, frailty leads to an increased risk of adverse events such as falls, impaired cognition, loss of independence, hospitalisation, and death, regardless of the metric used [54–56]. Advances in understanding these contributing biologic factors will lead to the development of multimodal non-pharmacological and pharmacological interventions with the goal to either prevent the decline or maintain a higher functionality of the overall system [57, 58].

Most evidence suggests that frailty in OPLWH is similar to that observed in physiologic aging [59]. The AGEHIV Cohort demonstrated a similar distribution in the FP parameters in the seropositive cohort compared with well-matched controls, although the prevalence of persons meeting both prefrailty and frailty criteria was higher in OPLWH [35].

In addition to the hallmarks of aging which contribute to the development of frailty in PLWH, the concurrence of inflammaging with immunosenescence, driven by ongoing immune activation due to persistent low-grade HIV replication even in fully HIV suppressed individuals, is the important additional element [60, 61]. Dysregulated chronic inflammation, especially of the innate immune system, is a consistent driving mechanism in clinical studies examining the biological determinants of frailty in PLWH [62–64]. Recently, epigenomic dysregulation was also suggested to play a role in the progression of frailty in PLWH [65].

Several clinical determinants particular to PLWH modulate progression to frailty. Most importantly, early control of HIV replication by cART, by achieving consistent virological suppression leading to CD4 + T-cell recovery, decreases the risk of developing frailty [66]. Guaraldi et al. demonstrated an inverse relationship between the CD4/CD8 T-cell ratio and the risk of frailty in the MMHC cohort [67]. A similar relationship between frailty and immune reconstitution was shown in a Spanish cohort [68]. Co-infections, including CMV and HCV, and microbiome translocation, which contribute to the persistent immune activation, are also cofactors in the development of frailty [69, 70]. These clinical attributes underline the importance of the immune-inflammatory parameters’ contribution to the development of frailty [71, 72]. Although frailty appears to occur earlier in PLWH, it fundamentally represents the same dysregulated state of physiological functions and decreasing functional reserve resulting in adverse events occurring in the elderly [73].

### Interactions between frailty, sarcopenia and functional status

Sarcopenia is recognized as an important geriatric syndrome in both the general population and in PLWH [74, 75]. Frailty and sarcopenia share common characteristics of reduced muscular strength, mass, and morphologic changes suggesting that a bi-directional relationship is likely to coexist [76, 77]. Normal aging results in significant negative changes to body composition, affecting both lean mass and fat mass, with important impact on muscle function. Sarcopenia, as now formally defined, is characterized by low skeletal muscle mass, and poor muscular strength and performance [78]. While common in older age, sarcopenia can occur at a younger age in PLWH and is more common in PLWH compared to people who “age normally” (24.1% vs 11.1%, respectively). The odds of being sarcopenic is over sixfold higher in PLWH compared to age, sex, BMI, and ethnicity-matched HIV uninfected individuals [75]. PLWH have a reduced muscle mass and function compared to seronegative controls [79]. Important contributing factors include low BMI, toxicity of certain antiretroviral drugs, and effects of chronic immune activation and chronic inflammation [79]. However, early signs of muscle mass loss are present in mid-life even with complete viral suppression and CD4 reconstitution [80]. Abnormal muscle morphologic changes (common in both sarcopenia and frailty), most notably intramuscular fat infiltration, is accelerated upon initiation of antiretroviral medication [81]. Even though thigh cross-sectional area was greater in PLWH, muscle density was significantly lower and was accompanied by more pronounced infiltration of intramuscular fat [82]. These abnormal body composition changes favor reductions in subcutaneous adipose tissue and greater infiltration of visceral and intramuscular fat stores that negatively impact muscle function through mechanical compression and release of proinflammatory cytokines [83–85].

The accumulation of subclinical damage to skeletal muscle that are among the chief indicators of frailty and sarcopenia in PLWH can give rise to other health deficits that are characteristic of both the FP and FI parameters. In a cross-sectional analysis of the Multicenter AIDS Cohort Study (MACS) Bone Strength Substudy, the odds of frailty was 4.5 × greater in the presence of sarcopenia [86]. The MACS demonstrated more rapid decline in gait speed trajectories, characteristic of both sarcopenia and frailty, in PLWH [87], and were accelerated by older age, lower nadir CD4 cell count and greater viral load. Similarly, grip strength decline was greater in PLWH, with a higher HIV viral load being an important risk factor [88].

Computed Tomography (CT) and Magnetic Resonance Imaging (MRI) are the gold standard methods to measure muscle quantity and quality. However, their cost and required expertise have resulted in their use being limited to research settings. Formal recommendations for sarcopenia screening have been suggested in specialty practice or primary care [89]. The simple to use Strength, Assistance with walking, Rising from a chair, Climbing stairs, and Falls (SARC-F) [90] questionnaire is recommended for screening for sarcopenia in clinical practice by the European Working Group on Sarcopenia in Older People (EWGSOP2) [78]. Dual X-ray Absorptiometry (DXA), if accessible, is now considered the standard imaging modality used to quantify fat-free mass. Bioelectrical impedance is an alternative affordable and relatively simple tool to estimate muscle mass but the accuracy of readings is affected by hydration status, extremely low muscle mass, and obesity. More recently, B-mode ultrasound has been studied to estimate skeletal muscle mass at the bedside [91]. Diagnostic algorithms are in development to diagnose sarcopenia simply and reliably, thereby allowing it to be used in routine clinical care alongside frailty screening and assessment.

As noted above, frailty is considered distinct from disability or co-morbidities with the FP. OPLWH may also have significant functional limitations which occur earlier than in uninfected individuals [92]. A study of PLWH found a strong association between the FP and a standard measure of functional capacity, the Short Performance Physical Battery (SPPB; a composite measure of gait speed, chair stand, tandem balance) such that 46% of pre-frail and 94% of frail study participants had a score ≤ 10/12 indicative of physical function impairment [93]. Similarly, in a study of Spanish PLWH older than 55 year, of whom 15% were FP + , Branas demonstrated that 20% had a slow gait speed and more than 50% had significant functional impairment in the SPPB [68]. In a cross- sectional study of female PLWH, pro-inflammatory cytokines characteristic of the chronic inflammatory state in treated PLWH, (e.g., Interleukin-6, tumor-necrosis factor-alpha,) were shown to be associated with low SPPB scores of < 9 [94]. In a study of effectively treated PLWH > 50 years old, subjects had substantially lower gait speed, 30-s chair stand repetitions, grip strength, and 6-min walking distance compared to normative values of the general population [95]. The substantially low average gait speeds of participants were indicative of an incapacity to safely walk across the street. Thus, the complex interplay of deficits unique to PLWH including immunosuppression, viral load, and greater proinflammatory state can contribute both to greater degrees of frailty as well as decline in both ADLs and IADLs which safety, ability to function independently, and quality of life.

Physical inactivity may also contribute to functional decline [96]. PLWH accumulate little physical activity and are more inactive compared to people living with other chronic conditions [97]. Whether the underlying pathophysiological changes and combination of sarcopenia, frailty, and functional decline contribute to physical activity limitations warrants further investigation. It would be useful to understand whether the energetic cost of muscle activity is greater in PLWH versus uninfected persons, which may then contribute to accelerated decline in muscle phenotypic changes associated with frailty [98]. Indeed, PLWH may have greater biomechanical abnormalities in comparison to non-infected individuals which would make them more susceptible to rapid functional decline and increased risk of falls [99].

Taken together, there is a strong interaction between sarcopenia, frailty, and functional decline. Some investigators consider sarcopenia as a biologic substrate of frailty [76, 100]. This hypothesis remains to be tested in PLWH and is an important area of inquiry.

### Transitions between frailty states and the role of resilience

#### Transitions

The paradigm of aging is diversity. In the HIV setting, many OPLWH experience good physical and mental health, while others may become frail and require support to assist with disabilities. Frailty is potentially a preventable and reversible condition in patients with malnutrition, following chemotherapy, major surgery, or, in the HIV setting, in persons with untreated HIV disease whose frailty status improves after starting cART [67].

Studies in the general population have shown frailty to be a dynamic state [101, 102]. A better understanding of which factors predict a transition to a state of frailty, including those which are HIV-specific, may help to identify both at-risk PLWH and potentially modifiable risk factors. In the AGEhIV study of 598 PLWH and 550 HIV-negative closely matched controls older than 45, PLWH were at a twofold increased risk of progressing from a non-frail to a frail state. The risk was attenuated but persisted even after adjusting for waist-to-hip ratio, the number of pre-existent comorbidities, and depression [103]. In a US study among 1353 AIDS Linked to the IntraVenous Experience (ALIVE) participants with 9559 frailty transition assessments, 33% were HIV-infected. Younger age, higher education, employment, reduced comorbidity, HIV virologic suppression, elevated CD4 nadir (> 500cells/ml) and absence of a prior AIDS diagnosis were associated with reduced frailty progression and greater recovery from frailty. Being frail at two consecutive visits demonstrated the highest mortality risk [63]. Table 2 summarizes risk and protective factors for frailty progression and reversibility. Many investigators consider that frailty reflects biological age, as opposed to chronological age, that it can be objectively assessed [104], and should be used in clinical decision-making algorithms instead of chronological age.

**Table 2:** Protective and risk factors for frailty progression and reversibility

	**Risk factors associated with frailty progression**	**Protective factors associated with frailty reversal**
**Socio-demographic factors**		
Older age	+	
Younger age		+
Low aging satisfaction	+	
Older subjective age	+	
Female sex	+	
Higher education		+
Employment		+
**Anthropometric variables and lifestyles**		
Smoking pack years	+	
Waist to hip ratio	+	
Combined exercise-nutrition intervention		+
**HIV related variables**		
Duration of ART exposure	+	
HIV virologic suppression		+
CD4 nadir > 500		+
Absence of AIDS diagnosis		+
Cumulative exposure to zalcitabine (ddC)	+	
HIV duration > 20 years	+	

#### Resilience

Complementary to the construct of frailty is that of “resilience” which also affects health trajectories and indicates the ability to avoid or recover functional decline after stressful events [105]. Resilience has been conceptualized as a dynamic trajectory over time in which various functions, after a stressor event, lead to either recovery or decline into a new equilibrium [106]. Understanding the underlying nature of the “resilience mechanisms” (homeostatic mechanisms) and “accumulated damages” (frailty), as well as defining methods to assess them, is an active area of investigation [106, 107].

Accumulated subcellular damage emerges clinically when compensatory physiologic mechanisms are exhausted. Both physical and cognitive decline may therefore result from these interrelated mechanisms, one inducing and the other preventing damage. The interaction between damage and repair could explain why some individuals appear to be aging “faster” and investigations may point to mechanisms supporting an accelerated aging process [107].

The COVID-19 pandemic crisis, focusing on the psycho-social burden and economic impact associated with lockdown and prolonged isolation, particularly in older adults, and acting independently from the clinical complications of COVID-19 disease, can be seen as a societal-level stressor event that challenges resilience. In this setting, the interaction between resilience and frailty can be assessed in relation to the potential impact on quality of life. In a recent study conducted in 575 PLWH enrolled in the MMHC, frailty was assessed in 2019, prior to the onset of the pandemic, using a 37-Item FI. Resilience was then assessed after the the 1^st^ wave of COVID using the Connor Davidson Resilience Scale. These two measurements allowed for the identification of four frailty-resilience phenotypes: “fit/resilient”, “fit/non-resilient”, “frail/resilient” and “frail/non-resilient”, based on previously reported cut-offs for both scores. Interestingly, poor quality of life evaluated with the EQ-5D5L questionnaire was predicted by the “frail/non-resilient” (OR = 5.21, 95% CI: 2.62; 10.33) and “fit/non-resilient” (OR = 5.48, 95% CI: 2.8; 10.74) phenotypes, suggesting that the resilience construct is complementary to frailty in the identification of clinical phenotypes with different impacts on health-related quality of life [108].

#### Management strategies

Most OPLWH in high-income countries currently receive their healthcare in either specialized community-based HIV primary care or tertiary care-based, usually in Infectious Diseases Clinics. As their care needs have expanded to include non-HIV related issues, combined geriatric or aging-HIV clinics, HIV-metabolic clinics or HIV-rehabilitation programs have been set up to address the wider social, mental and health challenges affecting OPLWH [109].

The model of care adopted by many such clinics is based on geriatric principles of care of older adults. These include screening for frailty and other geriatric syndromes, with recommendations for adapting the Comprehensive Geriatric Assessment (CGA) model to those OPLWH at the greatest risk. The CGA is a validated multidimensional, interdisciplinary diagnostic process used to determine the medical, psychosocial, and functional capabilities of selected older adults [110].

In the general population, annual screening for frailty is recommended after the age of 70 [111]. The most recent EACS Guidelines [25] recommend annual screening for frailty in PLWH over 50 using the FRAIL Scale and referring those who screen positive for an in-depth geriatric assessment [20]. HIV clinics with established care programs for OPLWH currently use various metrics to screen for frailty. These include assessing gait speed and using the FRAIL Scale [20, 112, 113] and the Clinical Frailty Scale [114]. Several centers have published their experience in managing frail OPLWH. A US, tertiary care, academic-based HIV and aging clinic accepts all referrals of OPLWH and then performs interdisciplinary geriatric assessments to screen for the most vulnerable [21]. The Chelsea and Westminster Clinic in London screens OPLWH with the CFS and refers those who are more than Stage 5 (at least mildly frail) to a dedicated Geriatric HIV clinic [114]. The evidence does not support using one frailty screening measure over another. Clinics may choose to use the afore-mentioned FP or FI metrics to diagnose frailty. The latter can be easily determined if an electronic health record system is in place [115, 116]. The FP requires specialized equipment (hand-grip dynamometer and stopwatch to determine gait speed) and training of personnel. Some centers may choose to use adapted FP parameters but this approach can lead to differences in the prevalence of diagnosed frailty compared to using the standard diagnostic FP criteria [117].

In OPLWH diagnosed as frail, the therapeutic goals are the prevention and management of disability and comorbidities, as well as the assessment and treatment of geriatric syndromes [118]. There are ongoing management interventions for PLWH, which adapt successful elements of studies in older uninfected persons with high degrees of frailty. Awareness of the increased health risks associated with social isolation [119] is also important to maintain quality of life and may help prevent cognitive decline. This framework re-integrates HIV care into the primary care model and makes an important effort to involve OPLWH within their social network and encourage the establishment and access to local community supports.

In frail, older HIV-negative persons, the basic principles of care include an exercise program with a strength training component, protein-calorie supplementation if weight loss or undernutrition is present, assessment for polypharmacy [120], assessment and management of sarcopenia, evaluation and management of reversible causes of exhaustion (anemia, depression, hypothyroidism and B12 deficiency), and evaluation and supplementation of vitamin D if indicated [121]. Multicomponent frailty prevention programs have limited the progression of frailty and shown improvement in physical function and in some cognitive domains [122]. A CGA-based care plan can improve functional ability, reduce risk of institutionalization and delay the development of disability, reduce admission and hospital stay, and improve survival [123–125].

Ideally, a physiotherapist should be accessible or part of the interdisciplinary team. Short-duration exercise programs can positively impact frail older adults [126]. Programs involving different intensity exercises have also improved physical function of OPLWH [127]. Participation in yoga programs improves quality of life metrics in PLWH [128].

It is anticipated that encouraging basic science advances will offer specific pharmacotherapeutic options to help manage frailty and related conditions. Although no specific agent for treating sarcopenia is currently licensed, early phase clinical studies of myostatin agonists and androgen receptor agonists have generated considerable interest and will likely have an important impact in the future [129]. Similarly, basic science discoveries support the early phase development of agents specifically targeting frailty [130].

Use of e-health based programs are increasingly able to provide health information, personal advice, and reminders through smart-phones to encourage healthy behaviors and assist older persons to improve and maintain their functional independence. The evaluation of wearable sensors to detect frailty is ongoing and has gathered much interest [131]. An interesting model is the Ecological Momentary Assessment, which collects patient-reported outcome measures in real time [132]. This may be a step forward to integrate patient-reported outcomes into novel health care models. It is also important to remain vigilant that frailty not become a tool to support an ageist approach to health care delivery [133, 134].

## Conclusions

Geriatric syndromes including frailty are highly prevalent in OPLWH. These may impact a person’s goal to live a satisfying health span. Understanding, and managing, where possible, the biologic determinants of frailty, the effects of sarcopenia, and consideration of factors contributing to transitions between frailty states must be at the forefront of providing care to OPLWH. In turn, planning for models of comprehensive care for frail OPLWH will require a great effort in terms of logistic coordination and closer interactions between various disciplines. At the core of this patient-focused approach is the introduction of reliable assessment and management strategies for frail PLWH who represent the most vulnerable group in the HIV/AIDS continuum. The successful accomplishment of such cross-disciplinary collaboration will not only markedly enhance the care of aging PLWH but will also constitute a model of successful healthcare management that can be applied to the graying of the entire population.

## Data Availability

Not applicable.

## Abbreviations

cART: Combination antiretroviral therapy
CFS: Clinical Frailty Scale
CGA: Comprehensive Geriatric Assessment
CT: Computed Tomography
DXA: Dual X-ray Absorptiometry
EACS: European AIDS Clinical Society
EFS: Edmonton Frail Scale
FI: Frailty Index
FP: Frailty Phenotype
FRAIL scale: Fatigue, Resistance, Ambulation, Illness, and Loss of we
MACS: Multicenter AIDS Cohort Study
MRI: Magnetic Resonance Imaging
OPLWH: Older persons living with HIV
PLWH: People living with HIV
SARC-F: Strength, Assistance with walking, Rising from a chair, Climbing stairs, and Falls
SPPB: Short Performance Physical Battery
VACS-I: Veterans Aging Cohort Study Index
